# Effect of *Shenlingyigan* decoction on inflammatory factors related to liver injury regulated by TLR3 signaling pathway

**DOI:** 10.1016/j.heliyon.2024.e24611

**Published:** 2024-01-23

**Authors:** Xiaoli Liu, Jun Li, Zhen Yang, Yanping Shi, Hui Ji, Xia Li

**Affiliations:** a Department of Integrated Traditional Chinese and Western Medicine, Xi'an Children's Hospital, The Affiliated Children's Hospital of Xi'an Jiaotong University, Xi'an, China; b Xi'an Hospital of Traditional Chinese Medicine, Xi'an, China

**Keywords:** TLR3 signaling pathway, *Shenlingyigan* decoction, Acute liver injury

## Abstract

**Background:**

To investigate the therapeutic effect of *Shenlingyigan* decoction on acute liver injury. Further explored the mechanisms involved in the therapeutic properties of *Shenlingyigan* decoction by test several key proteins (TLR3, TRIF, TBK1, IRF3, IFNβ, IL-1 and IL-6) within the TLR3 signaling pathway.

**Methods:**

The mouse acute liver injury model group was established by pretreatment with D-GalN and Poly (I:C) induction. The acute liver injury mouse treatment groups were gavage with different doses of *Shenlingyigan* decoction for 3 days. The therapeutic effects of *Shenlingyigan* decoction were preliminarily evaluated using organ indices, tissue images, and HE staining. Furthermore, potential associated signaling pathways and target effects were predicted through network pharmacology. Western blot experiments were conducted to examine the expression of relevant proteins (TLR3, TRIF, TBK1, IRF3, IL-1, and IL-6). In addition, immunofluorescence assays were performed to assess the localization of IRF3 and IFNβ expression in the cytoplasm and nucleus. Finally, the effects of *Shenlingyigan* decoction on the expression of TLR3, TRIF, TBK1 and IRF3 genes were further studied by QT-PCR.

**Results:**

The liver organ index, the tissue photos and HE staining showed that *Shenlingyigan* decoction could reduce inflammation by decreasing the presence of inflammatory cells and downregulating the expression of IL-1 and IL-6. The result of network pharmacology showed 709 potential drug and disease overlapping targets. Toll-like receptor signaling pathway was related with these targets through KEGG analysis. Besides, TLR3, TBK1, IRF3, IL6, were important targets associated with viral hepatitis. Westernblot and Immunofluorescence analysis showed that *Shenlingyigan* decoction reduced the expression of TLR3 and TBK1 in mice with liver injury, while increasing the expression of IRF3. *Shenlingyigan* decoction does not significantly affect the expression of TRIF and IFNβ; however, it enhances the expression of IRF3 in the nucleus, consequently leading to increased expression of IFNβ in the nucleus. The results of QT-PCR showed that *Shenlingyigan* decoction could down-regulate the expression of TLR3, TRIF and TBK1 genes, and up-regulate the expression of IRF3 gene.

**Conclusions:**

*Shenlingyigan* decoction participated in immune responses by effecting the expression of TLR3 signaling pathway-related factors to treat the acute liver injury.

## Graphical abstract

**Figure:**
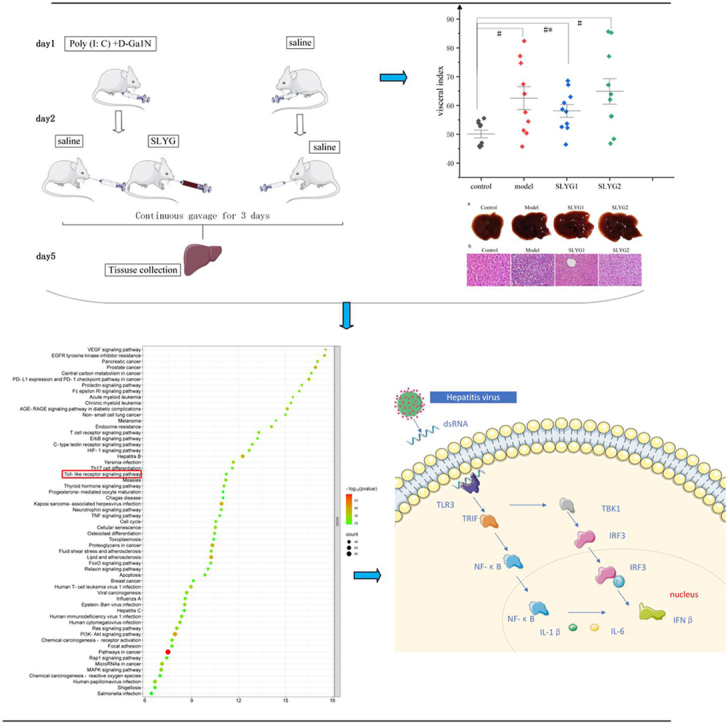

## 1 Introduction

Liver disease threatens global human health and is currently the leading cause of death. In America, acute and chronic liver disease, especially cirrhosis, consumes significant health care resources and costs [1]. Liver disease represents the third leading cause of premature mortality in the United Kingdom. Alarming statistics reveal that since 1970, the mortality rate attributed to liver disease has escalated fourfold in the UK, whereas all other significant causes of death have seen a decline in mortality rates. The data highlights a significant public health concern, and the need for effective measures to address the problem is paramount. The etiology of liver disease is multifaceted, with various factors such as lifestyle, genetics, infections, and environmental toxins contributing to its development. Therefore, targeted efforts should be made to prevent and manage liver disease through appropriate interventions such as lifestyle modifications, vaccination, and drug therapies. Immediate attention and intervention are necessary to curb this growing epidemic and improve public health outcomes in the UK [2,3]. There are about 300 million hepatitis patients in China and about 383,000 people die from liver cancer every year, accounting for 51% of the global liver cancer deaths% [4]. Therefore, curbing the increasing trend of global diseases caused by liver disease is of great significance to safeguarding the healthy lives of the people of the world.

The process of innate immune system recognizes pathogens was mediated by genome coding receptors [5]. The pattern recognition receptors (PRRs) are components of the bacterial membrane or the cell wall which are Lipoteichoic acid, lipopolysaccharide, and peptidoglycan [6]. PRRs like the toll-like receptor 3 (TLR3) of some innate systems could recognize dsRNA which was a common viral replication intermediate and a strong indicator of infection [7]. In addition, TLR3 is believed to recognize the hepatitis virus intermediate dsRNA, such as HAV, HBV and HCV, carried by themselves or produced during replication in infected cells, or endogenous dsRNA produced by damaged tissues or modulated dead cells [8,9]. TLR3 has implicated in autoimmune liver disease in animal models, but the role of TLR3 in human autoimmune diseases needs more studies to clarify [10]. The TLR3 signals the presence of extrinsic dsRNA. Upon RNA stimulation, TLR3 recruits the toll-interleukin 1 receptor domain (TIR)-containing adaptor molecule 1 (TICAM-1) and induces activation of IRF-3 followed by IFN-β promoter activation [11]. Interleukin (IL)-1 and Interleukin (IL)-6 was one of the cytokines induced by TLR3 that was essential to efficient response to tissue repair against trauma or infection (Fig. 1) [12].

**Fig. 1:**
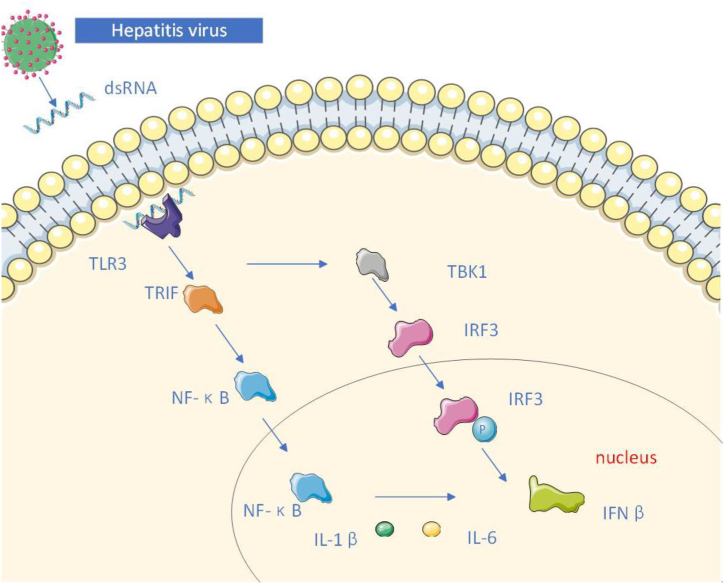
TLR3 signal pathway.

According to the research of Guedes and Yin [13,14] et al., TLR3 is associated with strong inflammation and liver damage. The viral load in the liver is positively correlated with the TLR3 gene, and activation of TLR3 in liver cells can induce the activation of IRF-3, thereby inhibiting the replication of hepatitis viruses (HBV, HCV, etc.). In addition, the study by Zou [15] et al. suggests that early activation of the TLR3/IFN pathway in the liver during HBV infection negatively regulates antiviral immune responses, which is detrimental to the clearance of HBV. This early activation may also be one of the reasons why immunocompetent adults develop chronic HBV infection after being infected with HBV. Therefore, it is of great significance to study the expression of TLR3 signaling pathway related factors in the early stage of liver injury for the treatment and prognosis of the disease.

Chinese medicine treatment of liver diseases has special advantages. Jun [16] et al. reviewed the use of traditional Chinese medicine extracts and derivatives, Chinese herbal formulae, and ethnic medicines for the treatment of liver injury. They found that traditional Chinese medicine exhibits characteristics of high safety, significant efficacy, and low toxicity. For instance, medicinal ingredients such as amygdalin, coumarins, and quercetin found in ancient ephedrine decoction and Liuwei Wuling tablets, as well as Andrographis paniculata, genistein, and Desmodium styracifolium (L.) Pers., mainly alleviate acute liver injury by improving oxidative stress and inflammatory response in the body.

The famous traditional Chinese medicine master, Professor Yang (Zhen Yang), was expert in treating liver disease. Professor Yang believes that children are the body of childish Yin and childish Yang, vulnerable to external evils, external evils directly lead to disease. Therefore, the pathogenesis of this disease is qi and yin deficiency, liver meridian blood heat, treatment was based on benefit qi and nourishing yin, and cooling blood detoxification. *Shenlingyigan* decoction, empirical prescription for treating acute liver injury of Professor Yang, had a significant effect in the treatment of acute liver injury, was consists of *Panax quinquefolius* L. *(Araliaceae), Ophiopogon japonicas (Asparagaceae), rehmannia root (Gesneriaceae), radix paeoniae alba (Paeoniaceae), ganoderma lucidum (Curtis), schisandra chinensis (Schisandraceae), Lithospermum erythrorhizon (Boraginaceae), Rubia cordifolia* L *(Rubiaceae), Lilium longiflorum Thunb. (Liliaceae), citrus chirocarpus (Rutaceae) and Glycyrrhiza glabra* L. *(Leguminosae)* [17,18]. As the complex mechanism of Chinese herbal compound components is not clear, this study aims to investigate the therapeutic effect of *Shenlingyigan* decoction on acute liver injury and elucidate the underlying mechanisms involved in its therapeutic properties.

## 2 Methods and materials

### 2.1 The preparation of SLYG

The formula of *Shenlingyigan* decoction (SLYG) is as follows: *Panax quinquefolius* L. *(Araliaceae), Ophiopogon japonicas (Asparagaceae), rehmannia root (Gesneriaceae), radix paeo-niae alba (Paeoniaceae), ganoderma lucidum (Curtis), schisandra chinensis (Schisandraceae), Lithospermum erythrorhizon (Boraginaceae), Rubia cordifolia* L *(Rubiaceae), Lilium longiflorum Thunb. (Liliaceae), citrus chirocarpus (Rutaceae) and Glycyrrhiza glabra* L. *(Leguminosae)*. The nomenclature of all plant species mentioned in this study adheres to the latest revision based on the “World Flora Online” (www.worldfloraonline.org) and MPNS (http://mpns.kew.org) databases. All the herbs included in the prescription were procured from Beijing Tongrentang Pharmacy, and their specific sourcing information is provided in Table 1.

**Table 1:** Ingredient information of *Shenlingyigan* decoction.

Full botanical plant names or zoological animal names	Local name	Dosing	Part	Species	Place of origin
*Panax quinquefolius* L.	Xi yangshen	8 g	Root	Araliaceae	Wisconsin,USA
*Ophiopogon japonicas*	Mai dong	8 g	Root	Asparagaceae	Ningxia, China
*rehmannia root*	Di huang	8 g	Root	Gesneriaceae	Anhui, China
*radix paeoniae alba*	Bai shao	10 g	Root	Paeoniaceae	Anhui, China
*ganoderma lucidum*	Ling zhi	8 g	whole plant	Curtis	Ningxia, China
*schisandra chinensis*	Wu wizi	10 g	Fruit	Schisandraceae	Anhui, China
*Lithospermum erythrorhizon*	Zi cao	10 g	Root	Bo-raginaceae	Xinjiang, China
*Rubia cordifolia* L	Qian cao	10 g	Root	Rubiaceae	shaanxi, China
*Lilium longiflorum Thunb.*	Bai he	8 g	Squamae	Liliaceae	Anhui, China
*citrus chirocarpus*	Fo shou	8 g	Fruit	Rutaceae	Zhejiang, China
*Glycyrrhiza glabra* L.	Gan cao	8 g	Root	Leguminosae	Anhui, China

Upon procurement, the medicinal materials were soaked in water (with 10 times the weight of the medicinal materials) for 30 min and boiled for 20 min. This process was repeated three times, and the obtained liquid was mixed and filtered. The resultant liquid was concentrated to a concentration of 3.5 g/ml and stored in a refrigerator. The dosage of *Shenlingyigan* decoction in the table is adult dose. Through the conversion of body surface area formula, the dosage of mice is set as 0.4 ml (1.4 g)/20 g. In order to compare the therapeutic effect of halving the dosage, we set the traditional Chinese medicine group as SLYG (1) is 0.4 (1.4 g) ml/20 g and SLYG (2) is 0.2 (0.7 g) ml/20 g.

### 2.2 Animals

Institute of Cancer Research (ICR) mice which were four-week-old and weighing 15–20 g, equally male and female, were purchased from Hunan Laike Jingda Experimental Animal Co. Ltd. (SCXK (Hunan) 2019-0004). The modeling drugs are as follows: D-GalN and Poly (I:C) Sigma-Aldrich (St. Louis, MO, USA).

The mice were offered food and water freely. We housed the mice in rooms with light and dark cycle every 12 h. Experimental schemes and animal nursing have the approbation of the Institutional Animal Care and Use Committee of Xi’an Children’s Hospital. All animals are treated humanely, and every effort is made to minimize animal suffering and population. Forfty mice were randomly divided into 4 groups, each mouse in the model group and in the SLYG (1,2) groups was injected intraperitoneally with 10 mg/mouse D-GalN, followed by 1 μg/mouse Poly (I: C). Each mouse in the control group was intraperitoneally injected with the same amount of normal saline. As show in Fig. 2. (1) The control group that was treated with saline. (2) The model group (Poly (I: C) + D-GalN) that was treated with saline, and (3) The three SLYG treatment groups (Poly (I: C) + D-GalN + SLYG) that was treated with a different dose of SLYG (1,2) on the second day after modeling.

**Fig. 2:**
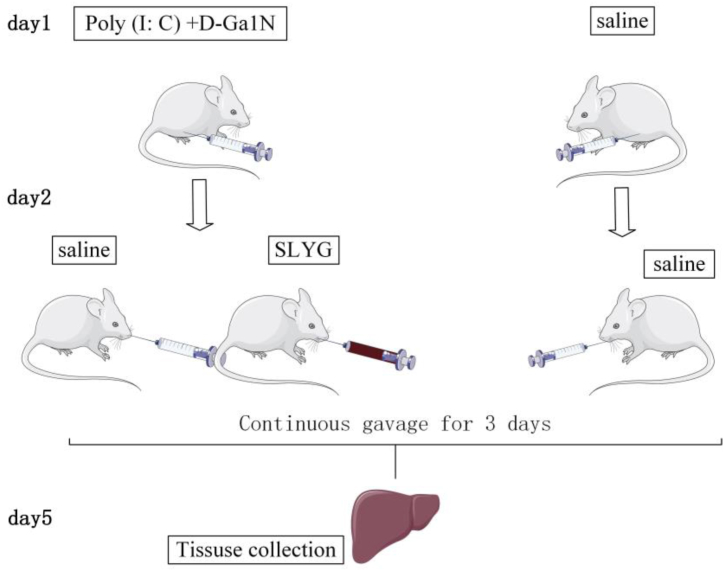
Model-making process of the animal experiment.

### 2.3 Organ index calculation

All mice received intragastric administration once a day for 3 days, and all mice were sacrificed. After killing the mice, the liver tissues were dissected and removed immediately. First, the liver in each group was photographed. The liver index was then weighed and recorded to calculate the liver index of the mice. The index was calculated according to the formula [19]: liver index = liver weight of mice (mg)/body weight of mice (g) × 100%.

### 2.4 Hematoxylin-eosin staining (HE)

The mice livers were washed with ice-cold saline after sacrificed, blotted with filter paper, and fixed by soaking in 10% neutral formalin. Each liver was dehydrated in different concentrations of C_2_H_5_OH, cleared with xylene, and embedded in paraffin. Sections were taken to 5 μm, baked at 70 °C for 5 min, dewaxed with xylene, and rehydrated with graded alcohol. 3 sections in each group were then stained with hematoxylin.

### 2.5 Network pharmacology analysis

The active chemical compounds of “the *Shenlingyigan* decoction which consist of *Panax quinquefolius* L., *Ophiopogon japonicas, rehmannia root, radix paeoniae alba, ganoderma lucidum, schisandra chinensis, Lithospermum erythrorhizon, Rubia cordifolia* L*, Lilium longiflorum Thunb, citrus chirocarpus,* and *Glycyrrhiza glabra* L.” were screened by the Traditional Chinese Medicine System Pharmacology (TCMSP) database [20]. The screening conditions were oral bioavailability (OB) ≥ 30% and drug-likeness (DL) ≥ 0.18. We converted each compound screened into canonical SMILES via PubChem. Subsequently, these SMILES were imported into SwissTargetPrediction, which could be used to predict potential targets of the compounds. The species were selected as “Homo sapiens,” and the probability >0.11 was used as the screening condition.

With “viral hepatitis” as the keyword, the disease-related targets were retrieved through the OMIM (https://omim.org/), and GeneCards – the human gene database (www.genecards.org/). Overlapping targets between potential targets of herb compounds and disease-related targets were considered potential targets for viral hepatitis. The protein-protein interaction (PPI) network was constructed by the STRING database (https://cn.string-db.org/) and visualized by Cytoscape 3.9.1 software. Kyoto Encyclopedia of Genes and Genomes (KEGG) pathway enrichment analysis was conducted by linking targets to the Database for Annotation, Visualization, and Integrated Discovery database (DAVID) (https://david.ncifcrf.gov/). Next, the picture of KEGG pathway enrichment was created using the online tools (http://bioinformatics.com.cn/).

### 2.6 ELISA

The prepared liver homogenate was used for ELISA to measure the expression level of Interleukin 6 (IL-6), and interleukin 1 (IL-1) (kits from Xiamen Lunchangshuo Biological Technology Co. LTD) according to the manufacturer’s protocol.

### 2.7 Western blot

The liver stored at −80 °C was weighed and added with RIPA lysis buffer (Biyuntian, China), the supernatant absorbed by centrifugation was the total protein of the liver after sufficient vortex lysis on ice. The samples’ protein concentration was test by BCA protein assay kit (Biyuntian, China). Sodium dodecyl sulfate-polyacrylamide gel electrophoresis (SDS-PAGE) (20 μg) were used to separate the samples and polyvinylidene fluoride (PVDF) membrane (Merck Millipore, USA) was used to transferred the proteins. The transferred membrane is incubated and bound with a primary antibody (TLR3 (rabbit polyclonal antibody, Abcam, USA) TRIF (rabbit polyclonal antibody, Abcam, USA) TBK1(rabbit polyclonal antibody, Abcam, USA) IRF3(rabbit monoclonal antibody, Abcam, USA) IFNβ (rabbit polyclonal antibody, Abcam, USA)), then the membrane is further incubated and bound with a second antibody conjugated with the appropriate horseradish peroxidase. Finally, protein bands were displayed using ECL chromogenic solution (Biyuntian, China).

### 2.8 Immunofluorescence assay (IF)

The liver sections in each group were place in citrate buffer solution (pH = 6.0), heat in microwave for 8 min, then cool to room temperature. 3% H_2_O_2_ added for 10 min at room temperature to inactivate endogenous enzymes. 5% sheep serum placed in wet boxes, sealed for 20 min at room temperature. After that incubated with the primary anti-nuclear factor antibodies (IRF3(rabbit monoclonal antibody, Abcam, USA) IFNβ(rabbit polyclonal antibody, Abcam, USA)) at 4 °C overnight. Then wash the sections for 3 times after conjugation with primary antibodies. They were incubated using the corresponding secondary antibody labeled with Cy3 (Sulfo-Cyanine3). Cell nuclei were stained with (DAPI 4’,6-diamidino-2-phenylindole). Suitable positive controls were utilized. Finally, the neutral gum was sealed, and the image was observed and preserved under an optical microscope (Leica, Germany).

### 2.9 Real-time quantitative RT-PCR

For total RNA extraction from tissue, a small portion of liver tissue sample was removed, frozen in liquid nitrogen and homogenized. 500 μl Trizol was added to the cell sample. To the 1.5 ml EP tube containing Trizol, 100 μl chloroform was added, mixed by shaking, and then allowed to stand for 5 min. The tube was centrifuged at 12,000 rpm and 4 °C for 10 min. The upper colorless and transparent aqueous phase was transferred to another clean 1.5 ml EP tube. The RNA precipitate was washed with 1 ml of 75% ethanol, air-dried at room temperature for approximately 5–10 min, and then reverse transcribed into cDNA. After the reaction was detected using PerfectStart Green qPCR SuperMix (Full Gold, AQ601-04), real-time fluorescence quantitative PCR instrument (ABI, Q1) was used for RT-PCR. The expression level of mRNA was calculated using the 2^-ΔΔCt method, with GAPDH gene as the internal reference. T-test was used to evaluate the difference in expression. The primer sequences are shown in Table 2.

**Table 2:** The primer sequences.

Primer name		Primer Sequence (5′–3′)
TLR3(NM_001357316.1)	Forward primer	TGCGCATATCACAGGCTGAA
	Reverse primer	GGTTCAGTTGGGCGTTGTTC
TRIF(NM_173394.3)	Forward primer	GCAGTACCACTTCCCAGCTAAC
	Reverse primer	GGCACCTTACCATTCGGTGA
TBK1(NM_019786.4)	Forward primer	TACGGTGGCTGGTTGAACTG
	Reverse primer	GGCTCATTGCTTTTGTGGCA
IRF3(NM_016849.4)	Forward primer	CCCAATTCCTCCCCTGGCTA
	Reverse primer	CTTCTTTCCGGTTCAGGGCT

### 2.10 Statistical analysis

Data differences in the icons are represented by the mean ± standard error (SEM) for each group. Analysis of variance (one-way analysis of variance) and *t*-test were used to detect whether the difference between groups was significant. Unless otherwise stated, all statistical analyses were performed using Origin8.0 (Originlab, USA). P < 0.05 was considered statistically significant. P < 0.1 was considered statistical difference.

## 3 Results

### 3.1 The *Shenlingyigan* decoction could reduce inflammatory reaction in the injured liver

During the whole process of modeling and administering acute liver injury in mice, no death was observed. The liver visceral index (n = 10) of mice was calculated and showed that the liver visceral index was significantly increased in the model group compared to the control group. The liver visceral index of mice in SYLG1 group was evidently less than that in model group (Fig. 3). As showed in Fig. 4a, the liver tissue photos of mice in the model group exhibited signs of congestion and swelling, whereas no such phenomenon was observed in the control group. However, the injured livers in the SLYG (1,2) group demonstrated an improvement in terms of congestion and swelling compared to the model group. The results of HE staining showed that lots of inflammatory cells infiltrated the liver tissue of mice in the model group. The situation was improved in the SLYG (1,2) group (Fig. 4b).

**Fig. 3:**
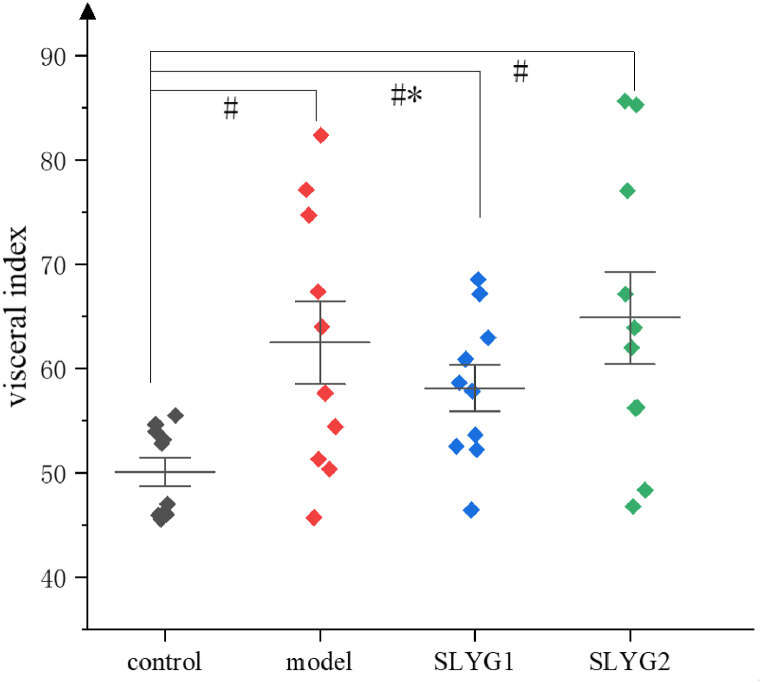
Visceral index (P < 0.1) * represents a significant difference from the model group, # represents a significant difference from the control group.

**Fig. 4:**
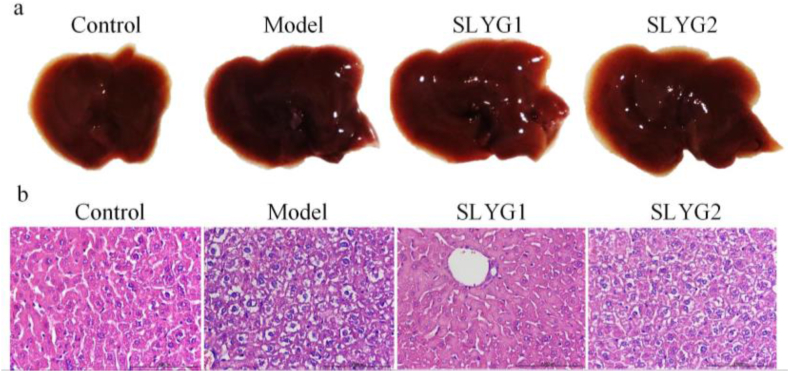
a. Liver tissue photos, b. Pictures of mice liver tissues (200×) in each group of HE staining.

### 3.2 Potential signaling pathways between *Shenlingyigan* decoction and viral hepatitis

We confirmed that *Shenlingyigan* decoction could treat acuate liver injury, and further explored its mechanism. The possible signaling pathway between the *Shenlingyigan* decoction and viral hepatitis was predicted based on network pharmacology. We retrieved 11 active ingredients of the *Panax quinquefolius* L., 9 active ingredients of the *Ophiopogon japonicas*, 9 active ingredients of the *radix paeoniae alba*, 8 active ingredients of the *rehmannia root*, 61 active ingredients of the *ganoderma lucidum*, 8 active ingredients of the *schisandra chinensis*, 12 active ingredients of the *Lithospermum erythrorhizon*, 17 active ingredients of the *Rubia cordifolia* L, 5 active ingredients of the *citrus chirocarpus*, 6 active ingredients of the *Lilium longiflorum Thunb.*, and 91 active ingredients of the *Glycyrrhiza glabra* L. from the TCMSP database. And then, we predicted 863 herb targets by SwissTargetPrediction. The Cytoscape software was used to analyze the effective components to obtain the target network diagram of the compound components (Fig. 5a), the shape size is positively correlated with the degree value. From Genecards and OMIM databases, we obtained 10,662 potential disease targets. We got 709 overlapping targets as showed in the Wayne diagram (Fig. 5b). According to the Molecular COmplex Detection (MCODE) score, we got the most closely connected regeions from the interactions of the 709 overlapping targets, including 57 overlapping targets (Fig. 5c). In addition, AKT1 gots the highest score in these targets. Through KEGG analysis of these targets, the first 60 relevant signal pathways were obtained and selected, as shown in the figure Fig. 5d, which included Toll-like receptor signaling pathway. We applied the criteria “Drugs & Compounds, Expression in Human Tissues, Pathways” to screen for target proteins associated with viral hepatitis. This screening process yielded 110 key targets, including TLR3, TBK1, IRF3 and IL6. The disease-associated network diagram was constructed using Cytoscape software for analysis as showed in Fig. 5e.

**Fig. 5:**
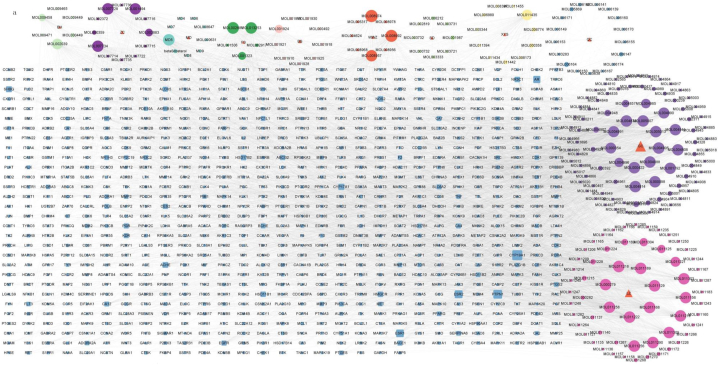

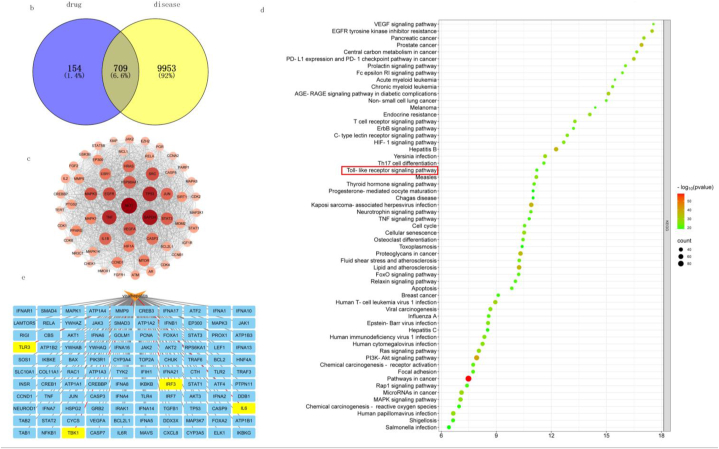
(a) Active ingredient-target network diagram of *Shenlingyigan* decoction, xys is *Panax quinquefolius* L., md is *Ophiopogon japonicas,* bs is *radix paeoniae alba*, sd is *rehmannia root*, lz is *ganoderma lucidum*, wwz is *schisandra chinensis*, zc is *Lithospermum erythrorhizon*, qc is *Rubia cordifolia* L, fs is *citrus chirocarpus*, bh is *Lilium longiflorum Thunb.*,gc is *Glycyrrhiza glabra* L. (b)Venn diagram of *Shenlingyigan* decoction and viral hepatitis; (c) The PPI network of key target protein of the intersection targets. The circular layout is arranged counterclockwise according to the node from light to dark and small to large. (d) KEGG pathway enrichment analysis. The larger the area of dots, the more the counts; the smaller the *P*-value, the bluer the dot color. Red box represent Toll-like receptor signaling pathway and Hepatitis B signaling pathway. (e). Yellow nodes represent TLR3, TBK1, IRF3 and IL6 respectively.

AKT1 is a key upstream kinase associated with NF-κB and IRF3 inflammatory signaling pathways in inflammation-related responses [21,22]. Notably, the search results in the PPI network of key target protein of the intersection targets showed that TLR3, TBK1, IRF3, IL6, were important targets associated with viral hepatitis (Fig. 5e). Toll-like receptors (TLRs) could recognize molecules unique to microbes so that distinguish self from non-self which play an important role in innate and adaptive immune responses. Toll-like receptor signaling pathway includes the Myd88-dependent pathway and the TRIF (Toll-interleukin-1 receptor domain-containing adapter inducing interferon-β)-dependent pathway. TRIF need its associated enzymes such as TBK1(TANK-binding kinase1) to induce translocation and activation of transcription factors such as NF -κ B (nuclear factor κ B), AP -1 (activator protein 1), and IRF3(interferonregulatory factor 3), besides, the TRIF/TBK1pathway is critical for IRF-3-mediated transcription [23]. The ligands of TLR3 were Poly I: C and dsRNA which were sourced from viruses and only works via the TRIF-dependent pathway [24,25]. Based on literature research and analysis results of network pharmacology, we speculated that the *Shenlingyigan* decoction could improve the acute liver injury caused by virus through modulation of the TLR3 signaling pathway.

### 3.3 The *Shenlingyigan* decoction can reduce the expression of IL-1 and IL-6 in the liver

IL-1 and IL-6 expression in the liver of mice in each group was tested by ELISA. The results in Fig. 6 showed that the expression of IL-1 in the model group and SLYG (1,2) groups increased significantly than that of control group. IL-1 in the SLYG (1,2) groups significantly decreased than that of the model group. There was a significant difference between the SLYG1 group and the model group. IL1 in SLYG2 group was also lower than that in model group, but the difference was not significant. The results in Fig. 7 showed that the expression of IL-6 increased a lot in the model group and SLYG (1,2) groups than that of control group. The expression of IL-6 decreased significantly in the SLYG1 group and SLYG2 group than that of the model group. The IL-6 expression in the SLYG1 group and the SLYG2 group did not have significantly difference (Fig. 7).

**Fig. 6:**
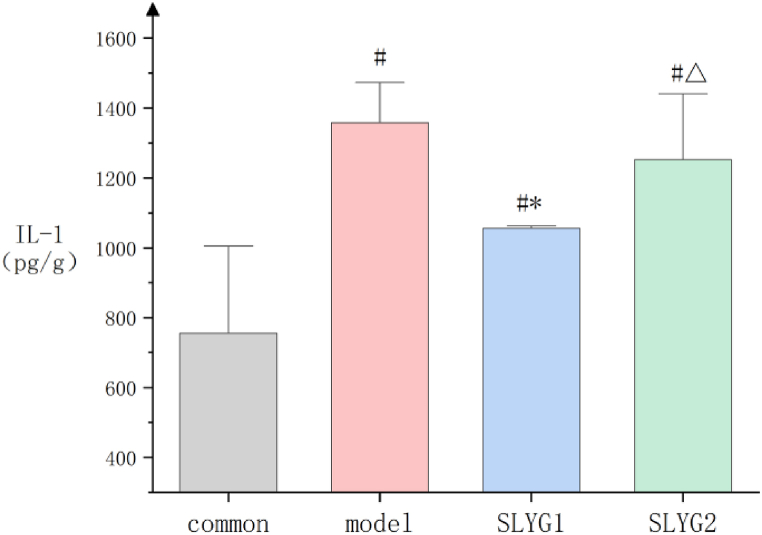
The expression of IL-1(P < 0.05). * represents a significant difference from the model group (n = 6); # represents a significant difference from the control group (n = 6); △ represents a significant difference from the SLYG1 group (n = 6).

**Fig. 7:**
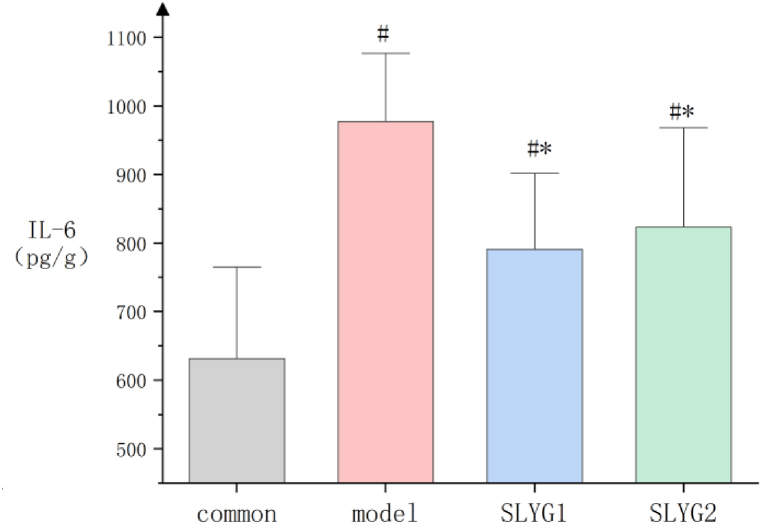
The expression of IL-6 (P < 0.05). * represents a significant difference from the model group (n = 6); # represents a significant difference from the control group (n = 6).

### 3.4 The *Shenlingyigan* decoction regulates the expression of TLR3 signaling pathway-related proteins

**Fig. 8:**
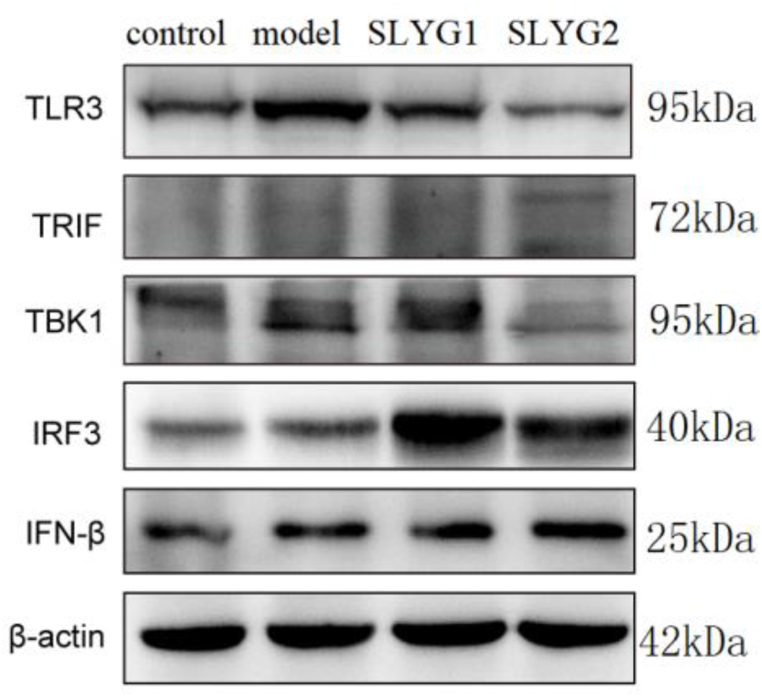
Effects of *Shenlingyigan* decoction on protein expression of vital genes related to TLR3 signaling pathway in response to acute liver injury.

To investigate the mechanism of action of *Shenlingyigan* decoction, we further examined several key proteins within the TLR3 signaling pathway. The results (Fig. 8) showed that compared to the control group, the expression levels of TLR3, TBK1, IRF3 and IFNβ were increased in the liver injury model group. In comparison to the model group, the expression levels of TLR3 and TBK1 were decreased in the SLYG1 and SLYG2 groups, whereas IRF3 expression was increased in both SLYG1 and SLYG2 groups, with no significant change observed in IFNβ expression. The expression level of TRIF was relatively low in all groups, with no significant differences observed. Additionally, we found that the downregulation of TLR3 and TBK1 was more pronounced in the SLYG2 group compared to the SLYG1 group, while the upregulation of IRF3 was more pronounced in the SLYG1 group compared to the SLYG2 group. These results indicate the therapeutic effects of *Shenlingyigan* decoction are associated with the TLR3 signaling pathway.

### 3.5 *Shenlingyigan* decoction changed the distribution of IRF3 and IFN-β between nucleus and cytoplasm

**Fig. 9:**
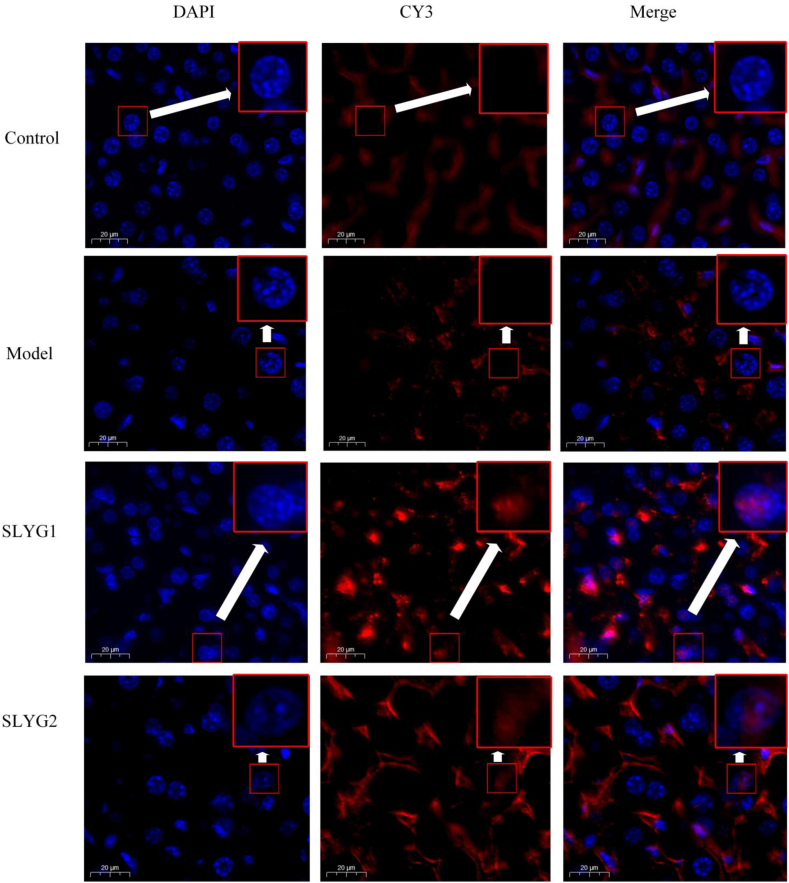
Effects of ***Shenlingyigan* decoction** on translocation of IRF3 into the nucleus. The blue represents the nucleus, and the red represents IRF3.

**Fig. 10:**
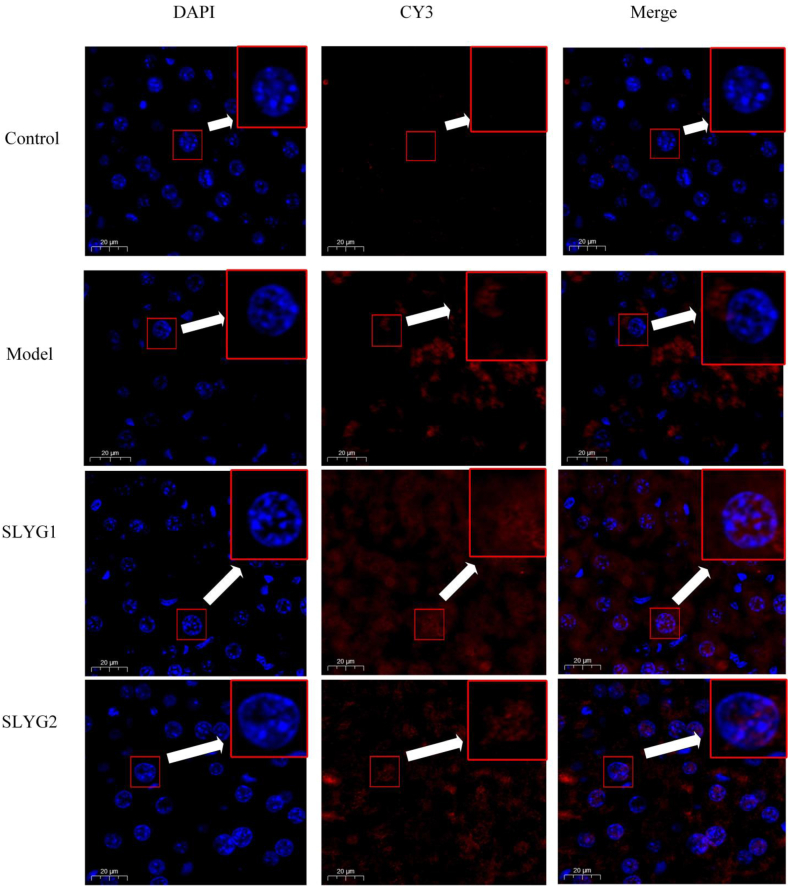
Effects of *Shenlingyigan* decoction on translocation of IFN-β into the nucleus. The blue represents the nucleus, and the red represents IFN-β.

The current study aimed to investigate the impact of *Shenlingyigan* decoction on the distribution of IRF3 and IFN-β between the nucleus and cytoplasm, utilizing immunofluorescence analysis through confocal microscopy. In the control group, IRF3 was primarily distributed in the cytoplasm in control group and model group; (Fig. 9). However, treatment with *Shenlingyigan* decoction was observed to cause a significant augmentation in the amount of IRF3 present in nuclei when compared to the model group. This phenomenon was more obvious in SLYG1 group than in SLYG2 group. Similarly, in the control group, IFN-β was detected to be distributed within the cytoplasm (Fig. 10). After modeling, the expression of IFNβ in the cytoplasm was significantly increased, but there was no significant expression in the nucleus. The expression of IFNβ in the nucleus was significantly increased in the SLYG1 group and the SLYG2 group when compared to the model group. Thus, the findings of the study support the hypothesis that *Shenlingyigan* decoction can promote the migration of IRF3 to the nucleus, as well as cause an increase in the distribution of IFN-β in the nucleus.

### 3.6 *Shenlingyigan* decoction regulates the expression of TLR3 signaling pathway-related genes

**Fig. 11:**
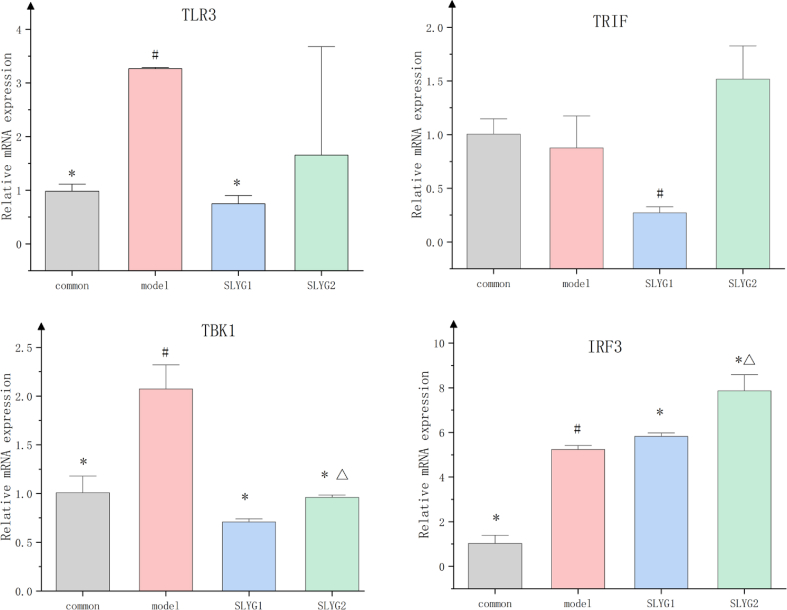
*Shenlingyigan* decoction regulates the expression of TLR3 signaling pathway-related genes mRNA levels in mice (n = 3) (P < 0.05). * represents a significant difference from the model group (n = 6); # represents a significant difference from the control group (n = 6); △ represents a significant difference from the SLYG1 group (n = 6).

In order to determine whether *Shenlingyigan* decoction intervenes in the changes of TLR3 signaling pathway-related gene levels, real-time quantitative PCR was used to detect the mRNA expression levels of TLR3, TRIF, TBK1, and IRF3 in liver tissue. The results are shown in Fig. 11. Compared to the control group, the expression level of TLR3 in the model group was significantly increased. In comparison to the model group, the expression level of TLR3 in the SLYG1 group was significantly decreased, and the SLYG2 group also showed a decrease, although there was no significant difference between the groups. Compared to the normal group, TRIF showed a significant decrease in expression in the SLYG1 group, with no significant differences between the other groups. TBK1 showed significantly higher expression in the model group compared to the normal group and SLYG group, with TBK1 in the SLYG1 group lower than that in the SLYG2 group, and the difference was significant. In comparison to the normal group, IRF3 showed a significant increase in expression in both the model group and SLYG group, with the SLYG group showing a significantly higher expression than the model group, and the SLYG2 group slightly higher than the SLYG1 group, with significant differences.

## 4 Discussion

TLR3 is believed to be able to recognize virus-carring or produced dsRNA in the replication process in infected cells, as well as endogenous dsRNA produced by injured tissue or apoptosis cells. TLR3 recognizes dsRNA from viruses and activates interferon regulatory factors through the TRIF-dependent pathway activated influential kinase like TBK1 to induce IRF3 to be rapidly phosphorylated in the nucleus and induces the secretion of IFN-α/β, which mediates the immune response of the body [26,27]. D-GalN is an RNA synthesis agent that can cause liver damage and macrophage infiltration [28]. Poly (I: C) can be specifically recognized by TLR3 and induce innate immune response and inflammatory response. It is a synthetic viral RNA and is widely used to simulate viral infection and establish models [29]. In this experiment, the acute liver injury model of mice was induced by Poly (I: C) and pretreated with d-galactosamine (D-GAlN). The body surface area method was used to calculate the dosage of *Shenlingyigan* decoction for mice ingavage as 0.4 ml/20 g to set this group SLYG1 group, and the dosage of 0.2 ml/20 g to mice SLYG2 group. To further study the therapeutic effect of *Shenlingyigan* decoction on acute liver injury.

Because the *Shenlingyigan* decoction is a compound, it is difficult to explain the specific components of the anti-inflammatory effect in the acute stage of liver injury. Luo et al. [30]. found that *Panax quinquefolius* L. capsules can improve the ultrastructural damage to the rat liver caused by electromagnetic radiation which associated with the reduction of oxidative stress and the protein expression of the phosphorylated ataxia mutant and the phosphorylated histone 2A variant. Xu et al. [31]. found that the *Panax quinquefolius* L. berry inhibits oxidative stress and the inflammation response through the caspase-3/-8/-9 signaling pathway mediated by TNF-α, and protects hepatocytes from acetaminophen-induced hepatotoxicity. Kitahiro and Tian et al. [32,33]. found that the *Ophiopogon japonicus* has antioxidant effect and can improve the antioxidant activity of macrophages. Wang [34]. found that the *Paeoniae radix alba* polysaccharides have an inhibitory effect on the immune inflammatory response of experimental autoimmune hepatitis model mice by inhibiting of the NF-κB signal path, reducing the hepatic infiltration of inflammatory CD8^+^ and CD4^+^ T cells, and decreasing the overexpression of the inflammatory cytokines IL-10, IL-6 and IL-2. Li et al. [35]. found that *Schisandrae Chinensis Fructus* could exert excellent effects on drug-induced liver injury by acetaminophen probably by reducing the TNF-α, IL-6, and IL-1β gene expression. Hassan and Guo et al. [36,37]. that *lucidum* extract could significantly inhibit abnormal elevation of serum triglyceride, aspartate aminotransferase, alanine aminotransferase, and can significantly protect the liver from excessive accumulation of liver lipids and pathological changes induced by alcohol. Xiong et al. [38]. found that *Rubia cordifolia* L. affects the phosphoinositide-3-kinase-protein kinase B and mitogen-activated protein kinase to. against liver cancer. Yoon et al. [39]. found that the *Lithospermum erythrorhizon* has anti-inflammatory effect and can treat fatty liver and liver fibrosis. In summary, the protective function of *Shenlingyigan* decoction may be related to its antioxidant and anti-inflammatory effects.

The result of network pharmacology showed that we got 709 overlapping targets of *Shenlingyigan* decoction and viral hepatitis. Toll-like receptor signaling pathway was related with these targets through KEGG analysis. Besides, TLR3, TBK1, IRF3, IL6, were important targets associated with viral hepatitis. Based on literature research and analysis results of network pharmacology, we speculated that the *Shenlingyigan* decoction could improve the acute liver injury caused by virus through modulation of the TLR3 signaling pathway.

In this study, the findings from the organ index analysis revealed an elevation in the liver organ index in mice with liver injury. However, the administration of *Shenlingyigan* decoction effectively reduced the liver organ index in these mice. The photographs of the liver in model group exhibited congestion and swelling. Notably, treatment with *Shenlingyigan* decoction demonstrated a significant improvement in liver congestion and edema. Furthermore, the results of HE staining indicated an increased presence of inflammatory cells in the liver tissue of mice with liver injury. Nevertheless, *Shenlingyigan* decoction exhibited the ability to reduce the number of inflammatory cells within the liver tissue of injured mice. ELISA experiment results showed an elevation of IL-1 and IL-6 in the liver injury group, and *Shenlingyigan* decoction could decrease the expression level of IL-1 and IL-6 in mice with liver injury. The SLYG1 group had a stronger effect in reducing IL-1 compared to the SLYG2 group, and the difference was significant. The SLYG1 group also had a stronger effect in reducing IL-6 compared to the SLYG2 group, but the difference was not significant. In summary, *Shenlingyigan* decoction has anti-inflammatory effect, and halving the dose also has anti-inflammatory effect.

Further studies on TLR3-related proteins revealed that, compared to the normal group, mice in the liver injury group showed increased expression of TLR3, TBK1, IRF3, and IFNβ. Compared to the model group, both doses of *Shenlingyigan* decoction reduced the expression of TLR3 and TBK1 in mice with liver injury, while increasing the expression of IRF3, with no significant change observed in IFNβ expression. The expression level of TRIF was relatively low in all groups, with no significant differences observed. Additionally, compared to the model group, both doses of *Shenlingyigan* decoction promoted the expression of IRF3 in the cell nucleus, thereby increasing the expression of IFNβ in the cell nucleus. To further determine the potential impact of *Shenlingyigan* decoction on the TLR3 signaling pathway-related gene expression levels, the RT-PCR results revealed that *Shenlingyigan* decoction can downregulate the expression of TLR3, TBK1, and TRIF genes while upregulating the expression of the IRF3 gene. Additionally, it was observed that SLYG1 exerts a stronger inhibitory effect on TBK1 compared to SLYG2, and SLYG1 exhibits a higher stimulatory effect on IRF3 compared to SLYG2, with these differences being statistically significant. Interestingly, there were no significant differences in the expression of TLR3 and TRIF between the SLYG1 and SLYG2 groups. These findings suggest that the mechanism of action of *Shenlingyigan* decoction is associated with the TLR3-TBK1-IRF3 signaling pathway. These findings are not surprising since all these factors are involved in triggering or controlling the inflammatory process [40,41].

Natural plant extracts are unique in the treatment of inflammatory diseases of the liver [42]. We speculate that there are multiple bioactive components in the *Shenlingyigan* decoction may be related to their antioxidation and anti-inflammatory effects just as in other TCM formulas. Due to the synergistic or additive effects that occur between the various bioactive components, the *Shenlingyigan* decoction plays an anti-inflammatory role by regulating factors related to the TLR3 signaling pathway in vivo. In addition, our findings demonstrate that both the SLYG1 group and SLYG2 group exhibited significant anti-inflammatory effects. This suggests that *Shenlingyigan* decoction retains its potent anti-inflammatory properties even at reduced dosage levels. Given that medications for children often require dosage adjustments when administered to adults, this study offers a crucial theoretical foundation for the use of *Shenlingyigan* decoction in pediatric medicine. However, further efforts are required to verify the exact molecular mode of action of *Shenlingyigan* decoction.

## 5 Conclusions

We demonstrated that *Shenlingyigan* decoction exhibits significant anti-inflammatory effects. In the early stages of acute liver injury, it effectively reduces inflammation by decreasing the presence of inflammatory cells and downregulating the expression of IL-1 and IL-6. *Shenlingyigan* decoction reduces the expression of TLR3 and TBK1 in mice with liver injury, while promoting the expression of IRF3. It does not significantly affect the expression of TRIF and IFNβ; however, it enhances the expression of IRF3 in the nucleus, consequently leading to increased expression of IFNβ in the nucleus. The results of QT-PCR showed that *Shenlingyigan* decoction could down-regulate the expression of TLR3, TRIF and TBK1 genes, and up-regulate the expression of IRF3 gene. These findings suggest that the therapeutic mechanism of *Shenlingyigan* decoction in liver injury is closely associated with the TLR3 signaling pathway. Additionally, the administration of reduced doses of *Shenlingyigan* decoction also demonstrates favorable anti-inflammatory effects, thus providing a theoretical basis for its use in pediatric medication.

## Ethics approval and consent to participate

The animal study protocol was approved by the Institutional Animal Care and Use Committee of Xi’an Children’s Hospital. (Permission number: 20220078).

## Funding

The authors would like to express their gratitude to Xi'an Children's Hospital for their support of this experimental study. This research was funded by Xi 'an Health Commission scientific research project; grant number 2022qn09 and Xi'an Health Commission Traditional Chinese Medicine Research Project; grant number SZL202205; 10.13039/100016067Shaanxi Provincial Administration of Traditional Chinese Medicine research Project; grant number 2021-ZZ-LC015.

## Availability of data and materials

The data used to support the findings of this study are available from the corresponding author upon request.

## CRediT authorship contribution statement

**Xiaoli Liu:** Writing – review & editing, Writing – original draft, Validation, Supervision, Software, Project administration, Methodology, Funding acquisition, Conceptualization. **Jun Li:** Writing – review & editing, Validation, Software, Resources, Methodology, Investigation, Data curation, Conceptualization. **Zhen Yang:** Visualization, Validation, Resources, Formal analysis. **Yanping Shi:** Visualization, Validation, Supervision, Project administration, Methodology, Funding acquisition, Formal analysis. **Hui Ji:** Investigation, Data curation. **Xia Li:** Investigation.

## Declaration of competing interest

The authors declare the following financial interests/personal relationships which may be considered as potential competing interests:Yanping Shi reports financial support was provided by 10.13039/100016067Shaanxi Provincial Administration of Traditional Chinese Medicine research Project. Xiaoli Liu reports financial support was provided by Health Scientific Research Talent Project of Xi’an Municipal Health Commission. If there are other authors, they declare that they have no known competing financial interests or personal relationships that could have appeared to influence the work reported in this paper.

## Figures and Tables

**figs1:**
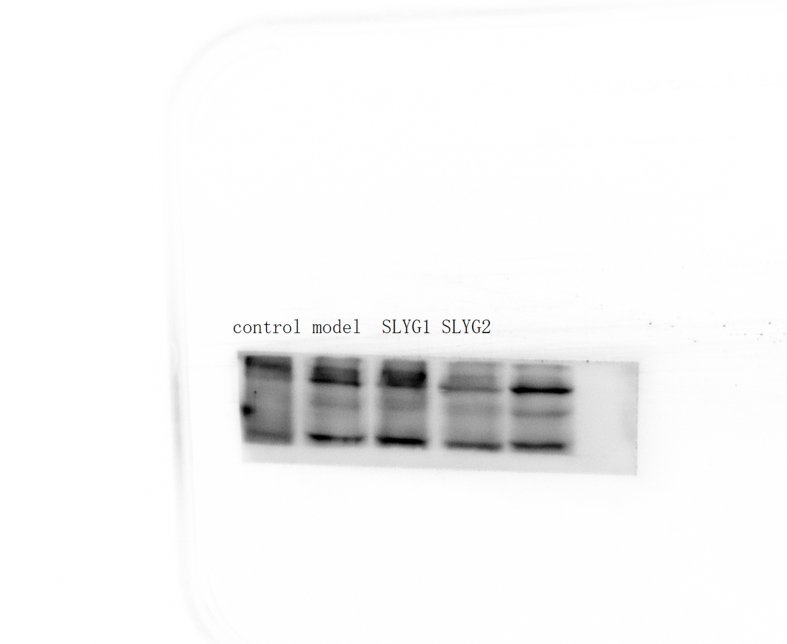


**figs2:**
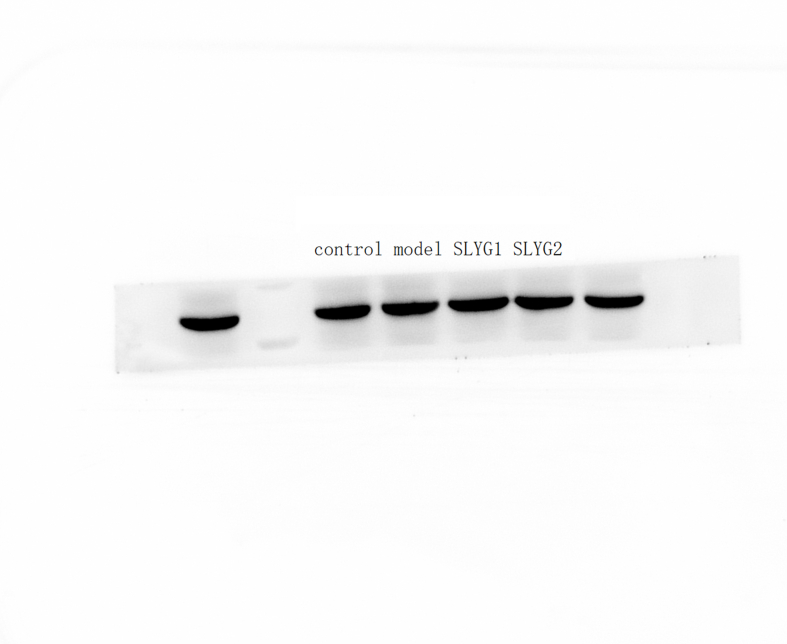


**figs3:**
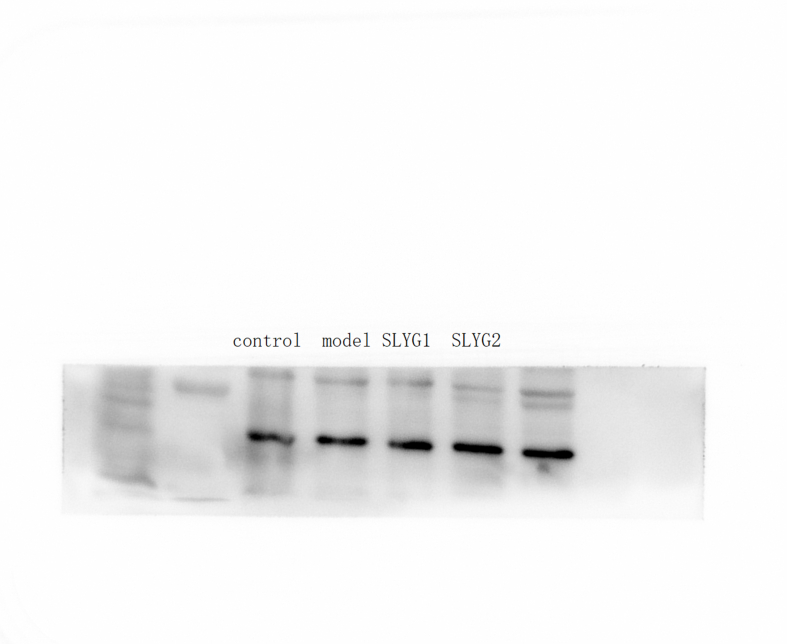


**figs4:**
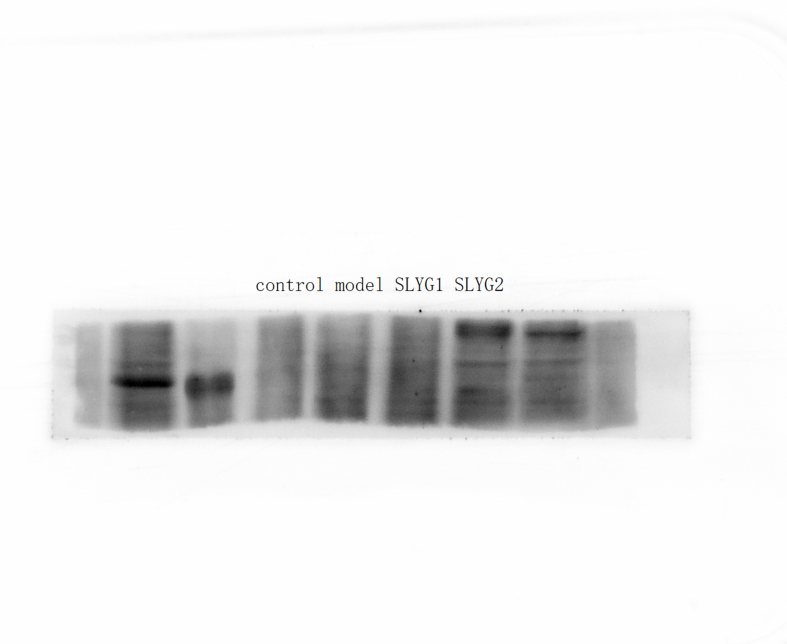


**figs5:**
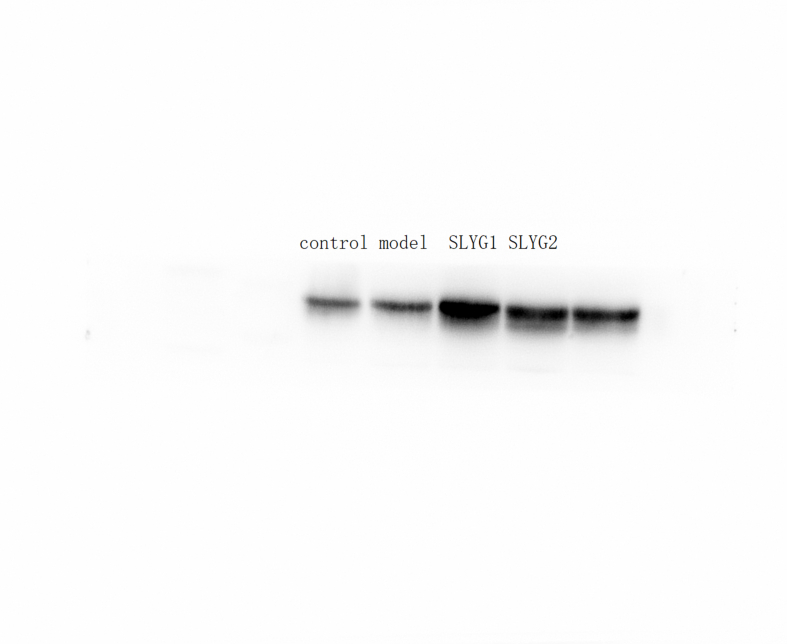


**figs6:**
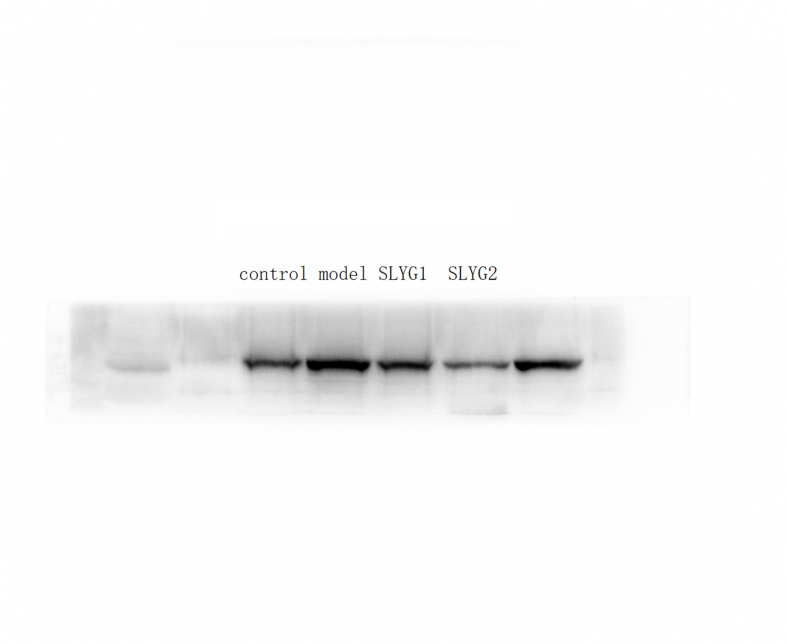


## Abbreviations

SLYG: *Shenlingyigan* decoction
TLR3: Toll-like receptor 3
TRIF: Toll-interleukin-1 receptor domain-containing adapter inducing interferon-β
TBK1: TANK Binding Kinase 1
IRF3: Interferon regulatory Factor 3
IFN-β: Interferon-β
IL-1: Interleukin 1
IL-6: Interleukin 6
ALT: Alanine aminotransferase
TBil: Total bilirubin
PPR: Pattern recognition receptors
TICAM-1: Toll-interleukin 1 receptor domain (TIR)-containing adaptor molecule 1
Poly (I: C): Polyinosinic-polycytidylic acid potassium salt
D-GAlN: d-galactosamine
ELISA: Enzyme-linked immunosorbent assays
HE: Hematoxylin-eosin staining
IF: Immunofluorescence
AKT1: RAC-alpha serine/threonine-protein kinase
